# Novel Leu-Val Based Dipeptide as Antimicrobial and Antimalarial Agents: Synthesis and Molecular Docking

**DOI:** 10.3389/fchem.2020.583926

**Published:** 2020-11-24

**Authors:** James A. Ezugwu, Uchechukwu C. Okoro, Mercy A. Ezeokonkwo, China R. Bhimapaka, Sunday N. Okafor, David I. Ugwu, Ogechi C. Ekoh, Solomon I. Attah

**Affiliations:** ^1^Department of Pure and Industrial Chemistry, University of Nigeria, Nsukka, Nigeria; ^2^Organic Synthesis and Process Chemistry, Council for Scientific and Industrial Research-India Institute of Chemical Technology, Hyderabad, India; ^3^Department of Pharmaceutical and Medicinal Chemistry, University of Nigeria, Nsukka, Nigeria; ^4^Department of Industrial Chemistry, Evangel University Akaeze, Enugu, Nigeria

**Keywords:** antimalarial, antimicrobial, benzenesulfonamide, Leu-Val dipeptide, *in silico* studies

## Abstract

The increase of antimicrobial resistance (AMR) and antimalarial resistance are complex and severe health issues today, as many microbial strains have become resistant to market drugs. The choice for the synthesis of new dipeptide-carboxamide derivatives is as a result of their wide biological properties such as antimicrobial, anti-inflammatory, and antioxidant activities. The condensation reaction of substituted benzenesulphonamoyl pentanamides with the carboxamide derivatives using peptide coupling reagents gave targeted products (**8a-j**). The *in silico* antimalarial and antibacterial studies showed good interactions of the compounds with target protein residues and a higher dock score in comparison with standard drugs. In the *in vivo* study, compound **8j** was the most potent antimalarial agent with 61.90% inhibition comparable with 67% inhibition for Artemisinin. In the *in vitro* antimicrobial activity, compounds **8a** and **8b** (MIC 1.2 × 10^−3^ M and 1.1 × 10^−3^ M) were most potent against *S. aureus*; compound **8a**, **8b**, and **8j** with MIC 6.0 × 10^−3^ M, 5.7 × 10^−4^ M, and 6.5 × 10^−4^ M, respectively, were the most active against *B. subtilis*; compound **8b** (MIC 9.5 × 10^−4^ M) was most active against *E.coli* while **8a**, **8b** and **8d** were the most active against *S. typhi*. Compounds **8c** and **8h** (MIC 1.3 × 10^−3^ M) each were the most active against *C. albicans*, while compound **8b** (MIC 1.3 × 10^−4^ M) was most active against *A. niger*.

**Graphical Abstract:**
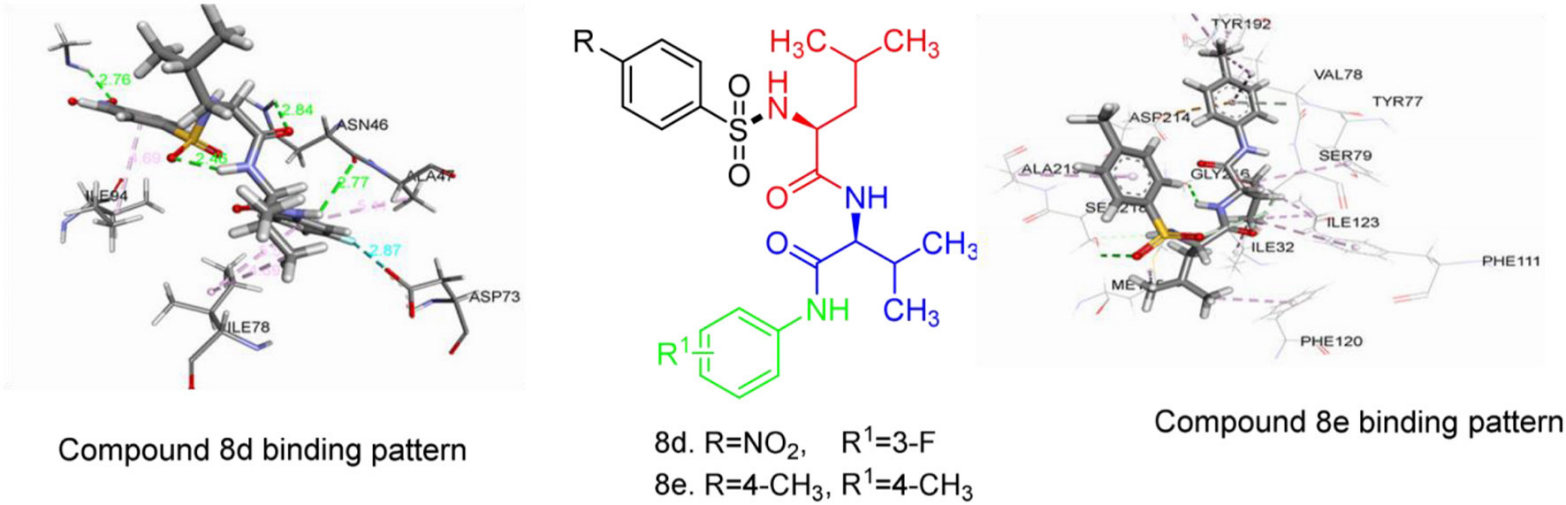
Graphical Abstract

## Introduction

Infectious diseases and their resistance to many available commercial drugs have remained the most challenging task for human existence. Sulfonamides consist of major drug components called sulfa drugs. The functional group –SO_2_NH_2_ in sulfonamides enables it to possess many pharmacological properties such as antibacterial (Qadir et al., 2015), antifungal (Jyothi and Madhavi, 2018), antimalarial (Maloy Kumar et al., 2018), anticancer (Abdelaziz et al., 2015), inhibitors of human carbonic anhydrase I (hCA I) and human carbonic anhydrase II (hCA II, Kilicaslan et al., 2016), anti-HIV (Jiao et al., 2010), and many others. Carboxamides are also ubiquitous functionality in medicate particles as pharmacophores (Montalbetti and Falque, 2005). Carboxamides have been accounted for as antimicrobial and antioxidant (Eze et al., 2019), carbonic anhydrase enzyme inhibitor and antioxidant (Deniz et al., 2019), anticancer (Kumar et al., 2009), anthelmintic (Ugwu et al., 2018a), antitubercular (Ugwu et al., 2014), antitrypanosomal (Ugwu et al., 2018b), and anti-inflammatory and analgesic (Ugwu et al., 2018c) agents.

Day and Greenfield (2004) reported that peptides are resourceful pharmacophores as they play important roles within the physical body and other organisms. As a result of good properties of peptides such as solubility, permeability, and bioavailability, many short peptide derivatives possess the ability to bind to membrane receptors (Qi et al., 2010; Thompson et al., 2012). Khavinson and Anisimov (2000) reported Lys-Glu based dipeptide as an antitumor agent. In 2004, Nitta et al. (2004) reported the neuroprotective effect of Leu-Ile. In the work of Kayser and Meisel (1996), Tyr-Gly was reported to reinforce the proliferation of peripheral blood lymphocytes. Peptides have also been reported as an antimicrobial (Jatinder et al., 2015), carbonic anhydrase I, II, IV, and XII inhibitor (Zehra Küçükbay et al., 2016; Küçükbay et al., 2019), antiplasmodial (Amit et al., 2015; Jatinder et al., 2015), and antihypertensive agent (Kitts and Weiler, 2003). In a continuation of our work, we synthesized novel Leu-Val dipeptide carboxamide scaffolds bearing sulfonamide moieties with potent antimalarial and antimicrobial properties. The quest for leu-val combination skeleton was as a result of antimalarial properties of ala-gly dipeptides (Ugwuja et al., 2019), antimalarial and antioxidant property of val-val dipeptides by Ezugwu et al. (2020), and also an antimalarial property of quinine derivatives containing some amino acid, dipeptide, or tripeptide (Panda et al., 2013).

### Experimental General

The chemicals and solvents used were purchased from Aldrich (Sigma-Aldrich) and AVRA Chemicals Pvt. Ltd. (Hyderabad, India) and used without purification. ^1^H-NMR and ^13^C-NMR spectra were recorded on Advance 300, 400, and 500 MHz spectrometers in DMSO-d_6_ using TMS as internal standard. FT-IR spectra were recorded on Thermo Nicollet Nexus 670 spectrometer. Mass spectra were obtained on Agilent LCMS instrument. HRMS were measured on Agilent Technologies 6510, Q-TOFLC/MS ESI-Technique. Melting points were determined in open glass capillary tubes on a Stuart melting point apparatus and are uncorrected. All experiments were carried out at Dr. B. China Raju's Laboratory, Organic Synthesis and Processing Chemistry Division, CSIR-Indian Institute of Chemical Technology, Hyderabad, India. All reactions were monitored by thin layer chromatography (TLC) on precoated silica gel 60 F_254_ (mesh); sports were visualized under UV light and in oven with Ninhydrin. Merck neutral aluminum oxide activated (60–325 mesh) was used for chromatography.

### Chemistry

#### General Procedure for the Synthesis of Substituted Benzenesulfonamoyl Pentanamides

Appropriate substituted benzenesulfonyl chloride (**1a-c**, 1.82 mmol) was added in portions for 1 h to an aqueous solution of L-leucine (1.5 mmol) containing sodium carbonate (Na_2_CO_3_, 1.82 mmol) at −5°C. The slurry formed was stirred at room temperature for 4 h (TLC (MeOH/DCM, 1:9 monitored). The mixture was acidified to pH 2 (Ugwu et al., 2014; Ezugwu et al., 2020).

#### General Procedure for the Synthesis of Carbmate Derivatives (6a-f)

To a solution of Boc-valine (3.0 g, 13.82 mmol) in dichloromethane (20 mL) was added triethylamine (20.7 mmol), EDC.HCl (16.0 mmol), HOBt (13.82 mmol) at 0°C, and substituted aniline (13.82 mmol) was added after stirring for 15 min. The resulting mixture was warmed to room temperature and stirred for 19–24 h (TLC monitored). Upon completion, the crude products were obtained after an aqueous work-up and purified by column chromatography (ethyl acetate/hexane = 5:95, Ezugwu et al., 2020).

#### Synthesis of Carboxamide Derivatives (7a-e)

Dichloromethane/trifluoroacetic acid (1:1%) was added to compounds **6a-c** and stirred at room temperature for 1 h (TLC monitored). The products were obtained after evaporating the solvent under reduced pressure. The solid TFA salts were precipitated on the addition of diethylether and dried (Ezugwu et al., 2020).

#### General Procedures for Synthesis of Leu-Val Dipeptides

To a solution of substituted benzenesulfonamoyl pentanamides (1.0 mmol) in dichloromethane (20 mL) was added triethylamine (20.7 mmol), EDC.HCl (16.0 mmol), HOBt (13.82 mmol) at 0°C, and carboxamide derivatives (**7a-e**, 1.0 mmol) were added after stirring for 15 min. The resulting mixture was warmed to room temperature and stirred for 19–24 h (TLC monitored). Upon completion, the crude products were obtained after an aqueous work-up and purified by column chromatography (ethyl acetate/hexane = 5:95, Ezugwu et al., 2020).

#### (S)-N-({(S)-1-[(4-Methylphenyl)amino]-3-methyl-1-oxobutan-2-yl})-4-methyl-2-(4-nitrophenylsulfonamido)pentanamide (8a)

Yield (0.315 g, 65.9%), light yellow solid, M.p = 172–17°C. FTIR (KBr, cm^−1^): 3,312, 3,261, and 3,194 (3NH), 2,965, 2,921 (C-H Aliphatic), 1,642, 1,640 (2C=O, amide), 1,533, 1,450 (C=C-Aromatic), 1,354 (SO_2_), 1,170, 1,090 (C-N**)**. ^1^H-NMR (400 MHz, DMSO) δ9.91 (s, 1H, NH of amide), 8.43 (dd, *J* = 8.5, 6.8 Hz, 1H, SO_2_-NH), 8.33 (d, *J* = 8.8 Hz, 2H, Ar-H), 8.12 (d, *J* = 8.6 Hz, 1H, NH of amide), 8.03 (dd, *J* = 8.9, 2.0 Hz, 2H, Ar-H), 7.44 (d, *J* = 8.4 Hz, 2H, Ar-H), 7.09 (d, *J* = 8.3 Hz, 2H, Ar-H), 4.04–3.95 (m, 2H, CH-C=O), 2.24 (s, 3H, CH_3_-Ar), 1.91–1.80 [m, 1H, CH-(CH_3_)_2_], 1.69–1.58 [m, 1H, CH-(CH_3_)_2_], 1.41–1.30 (m, 2H, CH_2_), 0.85 (d, *J* = 6.6 Hz, 3H, CH_3_), 0.79 (d, *J* = 6.5 Hz, 3H, CH_3_), 0.73 [dd, *J* = 6.6, 4.0 Hz, 6H (CH_3_)_2_]. ^13^C-NMR (101 MHz, DMSO) δ171.21, 169.85 (2C=O), 149.73, 147.17, 136.66, 132.73, 129.52, 128.72, 124.62, 119.62 (eight aromatic carbons), 58.78, 55.12, 42.29, 31.20, 24.34, 23.40, 21.59, 20.92, 19.45, 18.69 (10 aliphatic carbons). ESI-MS: m/z, 505 [M+H]^+^. HRMS-ESI: calcd. For C_24_H_32_N_4_O_6_S [M+H]^+^ 505.2121; Found 505.2125.

#### (S)-N-({(S)-1-[(4-Chlorophenyl)amino]-3-methyl-1-oxobutan-2-yl})-4-methyl-2-(4-nitrophenylsulfonamido)pentanamide (8b)

Yield (0.356 g, 71.6%), light yellow solid, M.p = 183–184°C. FTIR (KBr, cm^−1^): 3,300, 3,270, and 3,107 (3NH), 2,962, 2,871 (C-H Aliphatic), 1,643, 1,602 (2C=O, amide), 1,534, 1,497, 1,461 (C=C-Aromatic), 1,397, 1,348, 1,245, 1,207 (SO_2_), 1,164, 1,089, 1,013 (C-N**)**. ^1^H-NMR (400 MHz, DMSO) δ10.18 (s, 1H, NH of amide), 8.48–8.27 (m, 3H, SO_2_-NH + Ar-H), 8.10 (dd, *J* = 55.7, 1.2 Hz, 3H, NH of amide + Ar-H), 7.60 (d, *J* = 1.4 Hz, 2H, Ar-H), 7.35 (d, *J* = 0.8 Hz, 2H, Ar-H), 4.07 (t, *J* = 46.6 Hz, 2H, 2[C***H***-C=O]), 2.06–1.80 (m, 1H, CH-[CH_3_]_2_), 1.63 (d, *J* = 6.6 Hz, 1H, CH[CH_3_]_2_), 1.40–1.28 (m, 2H, CH_2_), 0.78 (dd, *J* = 34.3, 10.6 Hz, 12H [CH_3_]_4_).^13^C-NMR (101 MHz, DMSO) δ171.27, 170.29 (2C=O), 149.72, 147.16, 138.10, 129.10, 128.71, 127.39, 124.62, 121.11 (eight aromatic carbons), 58.88, 55.12, 42.26, 31.10, 24.34, 23.46, 21.60, 19.44, 18.67 (nine aliphatic carbons). ESI-MS: m/z, 525 [M+H]^+^. HRMS-ESI: calcd. For C_23_H_29_N_4_O_6_S [M+H]^+^ 525.1575; Found 525.1584.

#### (S)-N-({(S)-1-[(4-Isopropylphenyl)amino]-3-methyl-1-oxobutan-2-yl})-4-methyl-2-(4-nitrophenylsulfonamido)pentanamide (8c)

Yield (0.36 g, 72%), cream solid, M.p = 126–127°C. FTIR (KBr, cm^−1^): 3,330, 3,306, 3,101 (3NH), 2,962, 2,871 (C-H Aliphatic), 1,643, 1,609 (2C=O, amide), 1,533, 1,462 (C=C-Aromatic), 1,348, 1,246 (SO_2_), 1,163, 1,088, 1,017 (C-N). ^1^H-NMR (400 MHz, DMSO) δ9.90 (s, 1H, NH of amide), 8.38 (d, *J* = 8.9 Hz, 1H, SO_2_-NH), 8.33 (d, *J* = 8.9 Hz, 2H, Ar-H), 8.09 (d, *J* = 8.8 Hz, 1H, NH of amide), 8.03 (d, *J* = 8.9 Hz, 2H, Ar-H), 7.45 (d, *J* = 8.6 Hz, 2H, Ar-H), 7.15 (d, *J* = 8.5 Hz, 2H, Ar-H), 4.05–3.94 (m, 2H, 2[CH-C=O]), 2.82 (dt, *J* = 13.8, 6.9 Hz, 1H, CH-[CH_3_]_2_), 1.84 (dd, *J* = 13.7, 6.8 Hz, 1H, CH-[CH_3_]_2_), 1.69–1.57 (m, 1H, CH-[CH_3_]_2_), 1.41–1.26 (m, 2H, CH_2_), 1.16 (d, *J* = 6.9 Hz, 6H [CH_3_]_2_), 0.86–0.71 (m, 12H [CH_3_]_4_).^13^C-NMR (101 MHz, DMSO) δ171.21, 169.87 (2C=O), 149.74, 147.21, 143.91, 136.92, 128.72, 126.87, 124.63, 119.72 (eight aromatic carbons), 58.76, 55.08, 42.30, 33.36, 31.20, 24.43, 24.34, 23.48, 21.78, 21.61, 19.44, 18.71 (12 aliphatic carbons). ESI-MS: m/z, 533[M+H]^+^. HRMS-ESI: calcd. For C_26_H_36_N_4_O_6_S [M+H]^+^ 533.2434; Found 533.2445.

#### (S)-N-({(S)-1-[(3-Fluorophenyl)amino]-3-methyl-1-oxobutan-2-yl})-4-methyl-2-(4-nitrophenylsulfonamido)pentanamide (8d)

Yield (0.43 g, 89.6%), light yellow solid, M.p = 135–136°C. FTIR (KBr, cm^−1^): 3,302, 3,102 (NH), 2,963, 2,871 (C-H Aliphatic), 1,644, 1,610 (2C=O, amide), 1,536, 1,492, 1,442 (C=C-Aromatic), 1,348, 1,211 (SO_2_), 1,162, 1,088, 1,013 (C-N**)**. ^1^H-NMR (400 MHz, DMSO) δ10.22 (s, 1H, NH of amide), 8.38 (dd, *J* = 34.0, 8.6 Hz, 3H, SO_2_-NH + Ar-H), 8.13 (d, *J* = 8.2 Hz, 1H, NH of amide), 8.03 (d, *J* = 8.4 Hz, 2H, Ar-H), 7.55 (d, *J* = 11.5 Hz, 1H, Ar-H), 7.40–7.21 (m, 2H, Ar-H), 6.88 (d, *J* = 7.6 Hz, 1H, Ar-H), 4.12–3.87 (m, 2H, CH-C=O), 1.93–1.80 [m, 1H, CH-(CH_3_)_2_], 1.64 [d, *J* = 5.1 Hz, 1H, CH-(CH_3_)_2_], 1.35 (ddd, *J* = 17.3, 11.6, 6.7 Hz, 2H, CH_2_), 0.87–0.72 [m, 12H (CH_3_)_4_].^13^C-NMR (101 MHz, DMSO) δ171.32, 170.53 (2C=O), 163.77, 161.37, 149.72, 147.18, 140.86, 140.75, 130.91, 130.82, 128.72, 124.61, 115.32, 110.41, 110.20, 106.46, 106.19 (aromatic carbons), 58.84, 55.04, 42.25, 31.09, 24.33, 23.46, 21.57, 19.43, 18.63 (nine aliphatic carbons). ESI-MS: m/z, 509 [M+H]^+^. HRMS-ESI: calcd. For C_23_H_29_N_3_FO_4_S [M+H]^+^ 509.1870; Found 509.1870.

#### (S)-N-({(S)-1-[(4-Methylphenyl)amino]-3-methyl-1-oxobutan-2-yl})-4-methyl-2-(4-methylphenylsulfonamido)pentanamide (8e)

Yield (0.48 g, 96%), white solid, M.p = 191–192°C. FTIR (KBr, cm^−1^): 3,319, 3,267, 3,110 (3NH), 2,960, 2,910 (C-H Aliphatic), 1,643, 1,606 (2C=O, amide), 1,528, 1,456 (C=C-Aromatic), 1,377, 1,333, 1,244, 1,202 (SO_2_), 1,158, 1,088 (C-N). ^1^H-NMR (300 MHz, DMSO) δ9.93 (s, 1H, NH of amide), 7.99 (d, *J* = 8.5 Hz, 1H, SO_2_-NH), 7.89 (d, *J* = 9.0 Hz, 1H, NH of amide), 7.64 (d, *J* = 8.2 Hz, 2H, Ar-H), 7.48 (d, *J* = 8.4 Hz, 2H, Ar-H), 7.26 (d, *J* = 8.1 Hz, 2H, Ar-H), 7.11 (d, *J* = 8.3 Hz, 2H, Ar-H), 4.07 (t, *J* = 7.9 Hz, 1H, CH-C=O), 3.82 (td, *J* = 9.3, 5.3 Hz, 1H, CH-C=O), 2.26 (d, *J*=7.6 Hz, 6H, 2[CH_3_-Ar]), 1.86 (dt, *J* = 13.4, 6.7 Hz, 1H, CH-[CH_3_]_2_), 1.64–1.52 (m, 1H, CH[CH_3_]_2_), 1.30 (dd, *J* = 17.0, 8.4 Hz, 2H, CH_2_), 0.83–0.74 (m, 9H [CH_3_]_3_), 0.72 (d, *J* = 6.5 Hz, 3H, CH_3_). ^13^C-NMR (101 MHz, DMSO) δ171.64, 169.89 (2C=O), 142.78, 138.58, 136.73, 132.76, 129.70, 129.57, 127.09, 119.65 (eight aromatic carbons), 58.64, 55.18, 42.47, 31.44, 24.27, 23.47, 21.67, 21.37, 20.93, 19.50, 18.74 (11 aliphatic carbons). ESI-MS: m/z, 474 [M+H]^+^. HRMS-ESI: calcd. For C_25_H_36_N_3_O_4_S [M+H]^+^ 474.2427; Found 474.2424.

#### (S)-N-({(S)-1-[(4-Isopropylphenyl)amino]-3-methyl-1-oxobutan-2-yl})-4-methyl-2-(4-methylphenylsulfonamido)pentanamide (8f)

Yield (0.40 g, 75.7%), solid, M.p = 102–103°C. FTIR (KBr, cm^−1^): 3,313, 3,261, 3,105 (3NH), 3,050 (C-H-aromatic), 2,962, 2,871 (C-H Aliphatic), 1,645, 1,604 (2C=O, amide), 1,533, 1,458, 1,416 (C=C Aromatic), 1,378, 1,331, 1,204 (SO_2_), 1,158, 1,089 (C-N). ^1^H-NMR (300 MHz, DMSO) δ9.94 (s, 1H, NH of amide), 7.98 (d, *J* = 8.5 Hz, 1H, SO_2_-NH), 7.88 (d, *J* = 8.9 Hz, 1H, NH of amide), 7.65 (d, *J* = 8.0 Hz, 2H, Ar-H), 7.50 (d, *J* = 8.2 Hz, 2H, Ar-H), 7.26 (d, *J* = 7.9 Hz, 2H, Ar-H), 7.17 (d, *J* = 8.3 Hz, 2H, Ar-H), 4.08 (t, *J* = 7.8 Hz, 1H, CH-C=O), 3.82 (dd, *J* = 14.0, 8.8 Hz, 1H, CH-C=O), 2.83 (dt, *J* = 13.6, 6.8 Hz, 1H, CH-[CH_3_]_2_), 2.27 (s, 3H, CH_3_-Ar-H), 1.87 (dd, *J* = 13.4, 6.6 Hz, 1H, CH-[CH_3_]_2_), 1.68–1.49 (m, 1H, CH-[CH_3_]_2_), 1.42–1.26 (m, 2H, CH_2_), 1.17 (d, *J* = 6.8 Hz, 6H [CH_3_]_2_), 0.79 (dd, *J* = 9.8, 6.2 Hz, 9H [CH_3_]_3_), 0.72 (d, *J* = 6.4 Hz, 3H, CH_3_).^13^C-NMR (101 MHz, DMSO) δ171.64, 169.91 (2C=O), 143.94, 142.76, 138.62, 137.00, 129.70, 127.09, 126.90, 119.74 (eight aromatic carbons) 58.65, 55.18, 42.49, 33.37, 31.44, 24.44, 24.27, 23.47, 21.69, 21.34, 19.49, 18.75 (12 aliphatic carbons). ESI-MS: m/z, 502 [M+H]^+^. HRMS-ESI: calcd. For C_27_H_39_N_3_O_4_S [M+H]^+^ 502.2740; Found 502.2766.

#### (S)-N-({(S)-1-[(3-Fluorophenyl)amino]-3-methyl-1-oxobutan-2-yl})-4-methyl-2-(4-methylphenylsulfonamido)pentanamide (8g)

Yield (0.32 g, 64%), solid, M.p = 166–167°C. FTIR (KBr, cm^−1^): 3,327, 3,275, 3,185 (3NH), 2,962 (C-H Aliphatic), 1,680, 1,645 (2C=O, amide), 1,549, 1,491, 1,446 (C=C-Aromatic), 1,318, 1,212 (SO_2_), 1,157, 1,090 (C-N**)**. ^1^H-NMR (300 MHz, DMSO) δ10.27 (s, 1H, NH of amide), 8.05 (d, *J* = 8.4 Hz, 1H, SO_2_-NH), 7.89 (d, *J* = 9.0 Hz, 1H, NH of amide), 7.62 (dd, *J* = 16.4, 9.9 Hz, 3H,ArH), 7.38–7.23 (m, 4H, Ar-H), 6.89 (t, *J* = 8.1 Hz, 1H, Ar-H), 4.06 (t, *J* = 7.8 Hz, 1H, CH-C=O), 3.84 (dd, *J* = 14.3, 9.3 Hz, 1H, CH-C=O), 2.28 (s, 3H, CH_3_-Ar), 1.88 (dd, *J* = 13.5, 6.7 Hz, 1H, CH-[CH_3_]_2_), 1.58 (d, *J* = 6.2 Hz, 1H, CH-[CH_3_]_2_), 1.36–1.24 (m, 2H, CH_2_), 0.79 (dd, *J* = 12.0, 4.8 Hz, 9H [CH_3_]_3_), 0.73 (d, *J* = 6.5 Hz, 3H, CH_3_). ^13^C-NMR (101 MHz, DMSO) δ171.77, 170.61 (2C=O), 163.79, 161.39, 142.76, 140.94, 140.83, 138.62, 130.97, 130.88, 129.69, 127.09, 115.38, 110.43, 110.22, 106.50, 106.23 (aromatic carbons), 58.81, 55.09, 42.43, 31.27, 24.27, 23.47, 21.67, 21.35, 19.48, 18.71 (10 aliphatic carbons). ESI-MS: m/z, 478 [M+H]^+^. HRMS-ESI: calcd. For C_24_H_32_N_3_FO_4_S [M+H]^+^ 478.2176; Found 478.2181.

#### (S)-N-({(S)-1-[(4-Bromophenyl)amino]-3-methyl-1-oxobutan-2-yl})-4-methyl-2-((4-methylphenyl)sulfonamido)pentanamide (8h)

Yield (0.31 g, 81%), off-white solid, M.p = 140–142°C. FTIR (KBr, cm^−1^): 3,335, 3,269, 3,106 (3NH), 2,962, 2,873 (C-H Aliphatic), 1,687, 1,637 (2C=O, amide), 1,537, 1,489, 1,458 (C=C-Aromatic), 1,396, 1,315, 1,242 (SO_2_), 1,155, 1,088, 1,009 (C-N). ^1^H-NMR (400 MHz, DMSO) δ10.20 (s, 1H, NH of amide), 8.05 (d, *J* = 8.4 Hz, 1H, SO_2_-NH), 7.88 (d, *J* = 8.8 Hz, 1H, NH of amide), 7.65 (d, *J* = 8.2 Hz, 2H, Ar-H), 7.58 (d, *J* = 8.9 Hz, 2H, Ar-H), 7.49 (d, *J* = 8.9 Hz, 2H, Ar-H), 7.26 (d, *J* = 8.2 Hz, 2H, Ar-H), 4.07 (t, *J* = 7.9 Hz, 1H, CH-C=O), 3.83 (dd, *J* = 13.8, 8.9 Hz, 1H, CH-C=O), 2.27 (s, 3H, CH_3_-Ar), 1.88 (dp, *J* = 13.2, 6.6 Hz, 1H, CH-[CH_3_]_2_), 1.59 (tt, *J* = 13.2, 6.5 Hz, 1H, CH-[CH_3_]_2_), 1.38–1.24 (m, 2H, CH_2_), 0.89–0.76 (m, 9H [CH_3_]_3_), 0.73 (d, *J* = 6.5 Hz, 3H, CH_3_). ^13^C-NMR (101 MHz, DMSO) δ171.72, 170.39 (2C=O), 142.75, 138.61, 132.04, 129.68, 127.08, 126.10, 121.55, 115.41 (eight aromatic carbons), 58.67, 55.15, 42.44, 31.28, 24.27, 23.46, 21.68, 21.37, 19.49, 18.73 (10 aliphatic carbons). ESI-MS: m/z, 537 [M+H]^+^. ESI-HRMS: calcd. For C_24_H_32_N_3_BrO_4_S [M+Na]^+^ 560.1195; Found 560.1202.

#### (S)-N-({(S)-1-[(4-Chlorophenyl)amino]-3-methyl-1-oxobutan-2-yl})-4-methyl-2-((4-methylphenyl)sulfonamido)pentanamide (8i)

Yield (0.145 g, 61%), off-white solid, M.p = 177–179°C. FTIR (KBr, cm^−1^): 3,333, 3,272, 3,154 (3NH), 2,962 (C-H Aliphatic), 1,682, 1,643 (2C=O, amide), 1,540, 1,495, 1,459 (C=C-Aromatic), 1,397, 1,316, 1,247 (SO_2_), 1,156, 1,090, 1,012 (C-N). ^1^H-NMR (400 MHz, DMSO) δ10.16 (s, 1H, NH of amide), 8.01 (d, *J* = 8.5 Hz, 1H, SO_2_-NH), 7.86 (d, *J* = 9.1 Hz, 1H, NH of amide), 7.71–7.59 (m, 4H, Ar-H), 7.39–7.34 (m, 2H, Ar-H), 7.26 (d, *J* = 8.0 Hz, 2H, Ar-H), 4.07 (t, *J* = 7.9 Hz, 1H, CH-C=O), 3.83 (td, *J* = 9.4, 5.2 Hz, 1H, CH-C=O), 2.27 (s, 3H, CH_3_-Ar), 1.88 (tt, *J* = 13.7, 6.8 Hz, 1H, CH-[CH_3_]_2_), 1.59 (dt, *J* = 18.3, 6.6 Hz, 1H, CH-[CH_3_]_2_), 1.39–1.23 (m, 2H, CH_2_), 0.87–0.76 (m, 9H [CH_3_]_3_), 0.72 (d, *J* = 6.5 Hz, 3H, CH_3_). ^13^C-NMR (126 MHz, DMSO) δ171.73, 170.36 (2C=O), 142.75, 138.63, 138.16, 129.69, 129.16, 127.09, 126.10, 121.16 (eight aromatic carbons), 58.76, 55.10, 42.44, 31.30, 24.27, 23.48, 21.68, 21.37, 19.49, 18.74 (10 aliphatic carbons). ESI-MS: m/z, 494 [M+H]^+^, HRMS-ESI: calcd. For C_24_H_32_N_3_ClO_4_S [M+H]^+^ 494.1880; Found 494. 1879.

#### (S)-N-({(S)-1-[(Phenyl) amino]-3-methyl-1-oxobutan-2-yl})-4-methyl-2-(4-methylphenylsulfonamido)pentanamide (8j)

Yield (0.44 g, 91%), solid, M.p = 160–161°C. FTIR (KBr, cm^−1^): 3,307, 3,259, 3,194 (3NH), 3,058 (C-H-aromatic) 2,962, 2,921 (C-H Aliphatic), 1,643, 1,603 (2C=O, amide), 1,541, 1,446 (C=C Aromatic), 1,380, 1,324, 1,245, 1,205 (SO_2_), 1,159, 1,090 (C-N**)**. ^1^H-NMR (400 MHz, DMSO) δ10.01 (s, 1H, NH of amide), 7.99 (d, *J* = 8.5 Hz, 1H, SO_2_-NH), 7.87 (d, *J* = 9.0 Hz, 1H, NH of amide), 7.65 (d, *J* = 8.1 Hz, 2H, Ar-H), 7.60 (d, *J* = 7.8 Hz, 2H, Ar-H), 7.31 (t, *J* = 7.8 Hz, 2H, Ar-H), 7.26 (d, *J* = 8.0 Hz, 2H, Ar-H), 7.05 (t, *J* = 7.3 Hz, 1H, Ar-H), 4.10 (t, *J* = 7.8 Hz, 1H, CH-C=O), 3.83 (td, *J* = 9.2, 5.2 Hz, 1H, CH-C=O), 2.27 (s, 3H, CH_3_-Ar), 1.88 (dt, *J* = 13.4, 6.7 Hz, 1H, CH[CH_3_]_2_), 1.59 (dd, *J* = 12.5, 6.2 Hz, 1H, CH-[CH_3_]_2_), 1.39–1.23 (m, 2H, CH_2_), 0.80 (dd, *J* = 11.1, 6.3 Hz, 9H [CH_3_]_3_), 0.73 (d, *J* = 6.5 Hz, 3H, CH_3_). ^13^C-NMR (101 MHz, DMSO) δ171.68, 170.17 (2C=O), 142.77, 139.24, 138.61, 129.69, 129.23, 127.10, 123.84, 119.62 (eight aromatic carbons), 58.68, 55.17, 42.48, 31.41, 24.28, 23.47, 21.68, 21.36, 19.51, 18.73 (10 aliphatic carbons). ESI-MS: m/z, 460 [M+H]^+^, HRMS-ESI: calcd. For C_24_H_33_N_3_O_4_S [M+H]^+^ 460.2270; Found 460.2269.

### *In silico* Methodology

#### Physicochemical Properties

The drug-likeness of the synthesized compounds are shown in Table 1. The molecular parameters calculated include molecular weight (MW), partition coefficient (log P), hydrogen bond acceptor (HBA), hydrogen bond donor (HBD), topological polar surface area (TPSA), number of a rotatable bond (nRB), and molar refractivity (MR). The drug-likeness was determined using Lipinski's rule of five.

**Table 1:** Physicochemical properties of the compounds.

**Compds**.	**HBA**	**HBD**	**RC**	**nRB**	**nAC**	**NV**	**logP(o/w)**	**MR**	**TPSA**	**MW**
8a	4	3	2	11	0	1	4.45	13.40	150.19	504.61
8b	4	3	2	11	0	1	4.75	13.47	150.19	525.03
8c	4	3	2	12	0	1	5.30	14.32	150.19	532.66
8d	4	3	2	11	0	1	4.34	13.04	150.19	508.57
8e	4	3	2	10	0	0	4.82	13.32	104.37	473.64
8f	4	3	2	11	0	1	5.66	14.23	104.37	501.69
8g	4	3	2	10	0	0	4.71	12.96	104.37	477.60
8h	4	3	2	10	0	1	5.32	13.64	104.37	538.51
8i	4	3	2	10	0	0	5.11	13.39	104.37	494.06
8j	4	3	2	10	0	0	4.52	12.87	104.37	459.61

*TPSA, total polar surface area; NA, number of atoms; MW, molecular weight; HBA, hydrogen bond acceptor; HBD, hydrogen bond donor; NV, number of violations; nRB, number of rotatable bond. RC, ring compounds; nAc, number of acid; logP (o/w) means the logarithm of 1-octanol/water partition coefficient*.

#### Molecular Docking

In this study, proteins essential for malaria and bacterial infections were evaluated. The protein targets were plasmepsin II (PDB ID: 1SME) from *P*. *falciparum*, and (PDB ID: 5MMN) for antimalarial and antimicrobial studies, respectively, were obtained from the protein data bank.

Plasmepsin II from *P*. *falciparum* has been used as a novel target for antimalarial drug development because of its role as hemoglobin-degrading enzyme (Silva et al., 1996). Targeting the GyrB/ParE ATP-binding sites is an emerging approach in discovery of resistant bacteria. These ATP-binding sites located on bacterial DNA gyrase (5MMN) and topoisomerase IV, making them potent drug targets (Panchaud et al., 2017).

The 3D crystal structures of protein targets and co-crystallized ligands were from (https://www.rcsb.org/). The co-crystallized ligands were used to validate the docking protocols by redocking them into the active binding sites of the receptors. The structures of the molecules were drawn using ChemSketch. Further purification of the protein and ligands were furnished utilizing the discovery studio to erase various chains, the water of crystallization from the protein, and reduced the energy of the structures. Discovery Studio Visualizer, v16.1.0.15350 was used to envisage the interactions of the prepared ligands into the binding cavity of the protein receptors after docking. SwissADME was used to predict the physicochemical properties of the compounds.

#### Antimicrobial Evaluation

The microorganisms below were isolated clinically from University of Nigeria, Nsukka at Department of Pharmaceutical Microbiology and Biotechnology laboratory. S*taphylococcus aureus, Escherichia coli, Bacillus subtilis, Salmonella typhi, Candida albicans*, and *Aspergillus niger*.

##### Standardization of the test organism suspension

The standardization of the microorganisms was completed using 0.5 MacFaland turbid equivalent.

##### Control test (standard)

The standard antibiotics used were ciprofloxacin and fluconazole.

#### Experimental

Into a sterile petri dish containing a suspension of the stock (4 mL, 50 mg/mL) was added double strength sterile molten agar (16.0 mL) and mixed thoroughly to obtain 1 mg/mL solution. Lower concentrations (0.1–0.9 mg/mL) were calculated from the equation C_1_V_1_= C_2_V_2_. The plates were allowed to gel and divided into seven parts. The test microbes were patterned on the plates, labeled, and kept in an incubator at 37°C for 24 h and 35°C for 48 h, respectively, for antibacterial and antifungal activities. Further incubation of the plates for 24 h at 37°C and 48 h at 25°C, respectively, used to test for bactericidal and fungicidal activities.

### *In vivo* Anti-malaria Test

#### Experimental Design and Treatment of Mice

The antiplasmodial activity were assessed by adopting the methods of Okokon and Nwafor (2009) and Ezugwu et al. (2020).

## Results and Discussion

### Chemistry

**Figure 1:**
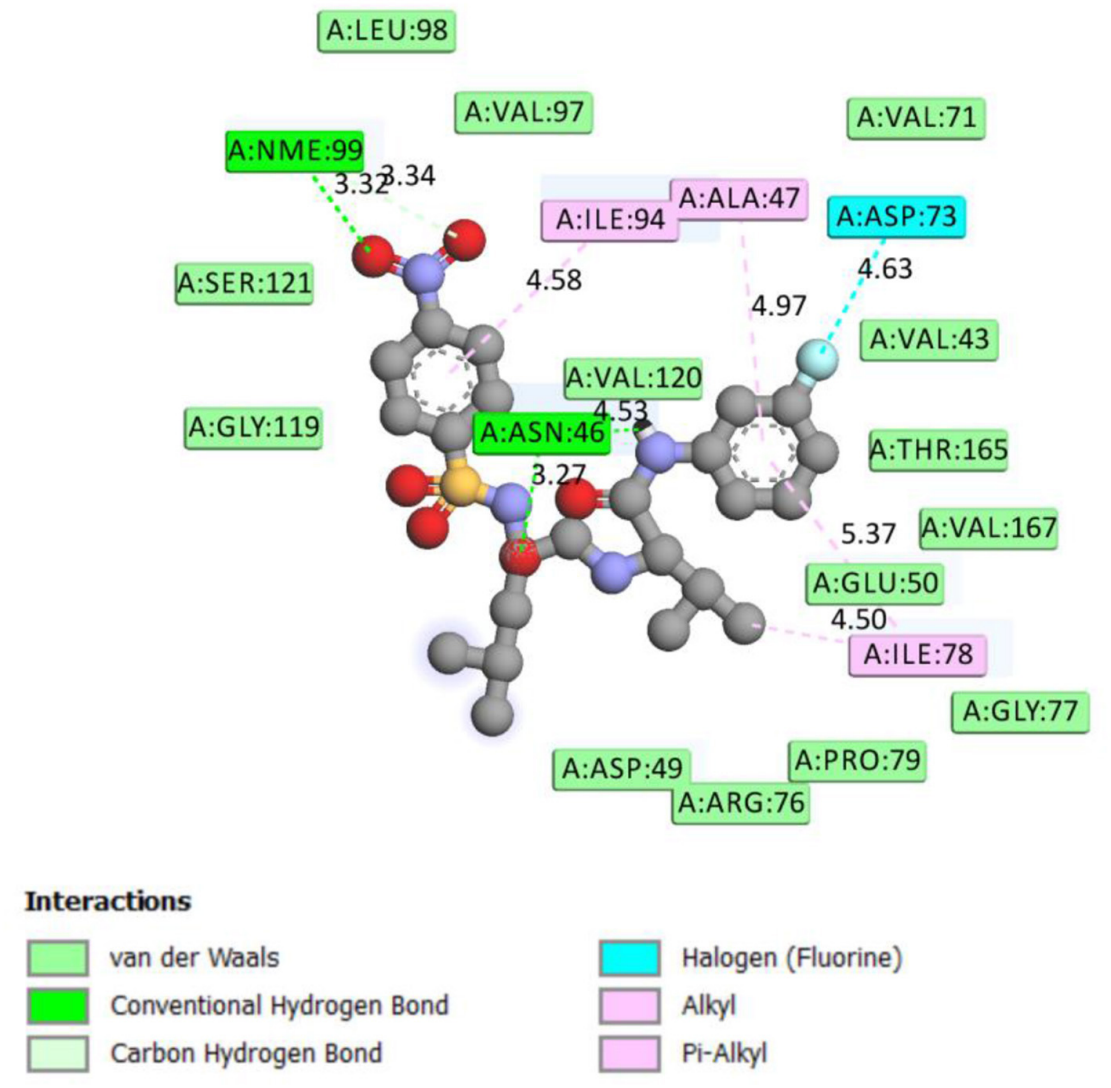
2D representation of binding interaction of compound 8d and the amino acid residues of 5MMN.

**Figure 2:**
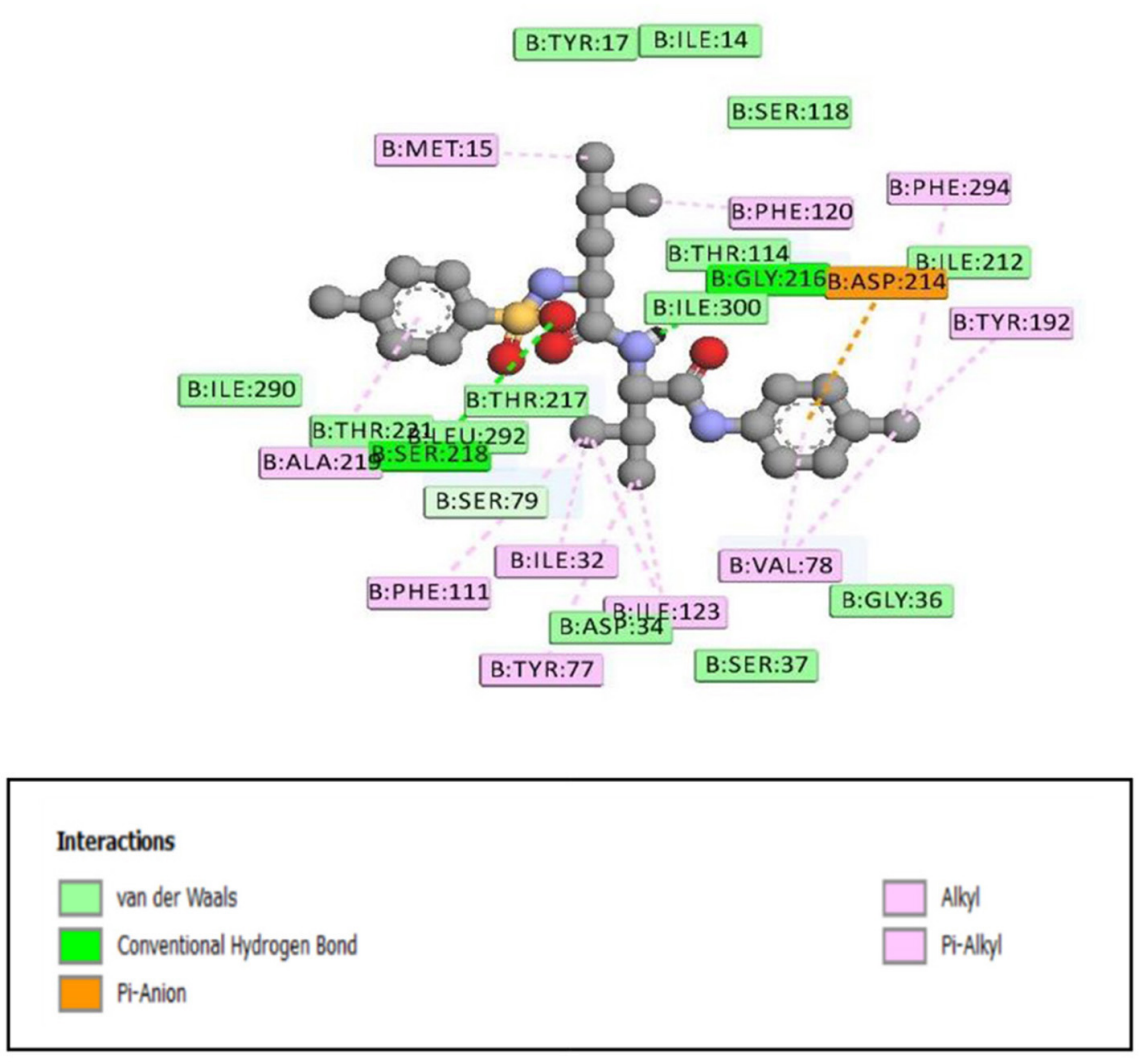
2D representation of binding interaction of compound 8e and the amino acid residues of 1SME.

**Scheme 1:**
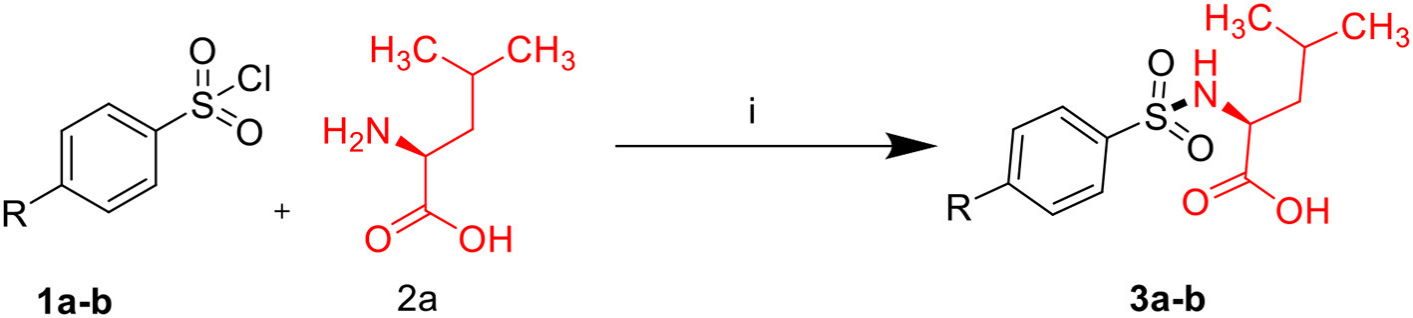
Synthesis of compounds (3a–b).

**Scheme 2:**
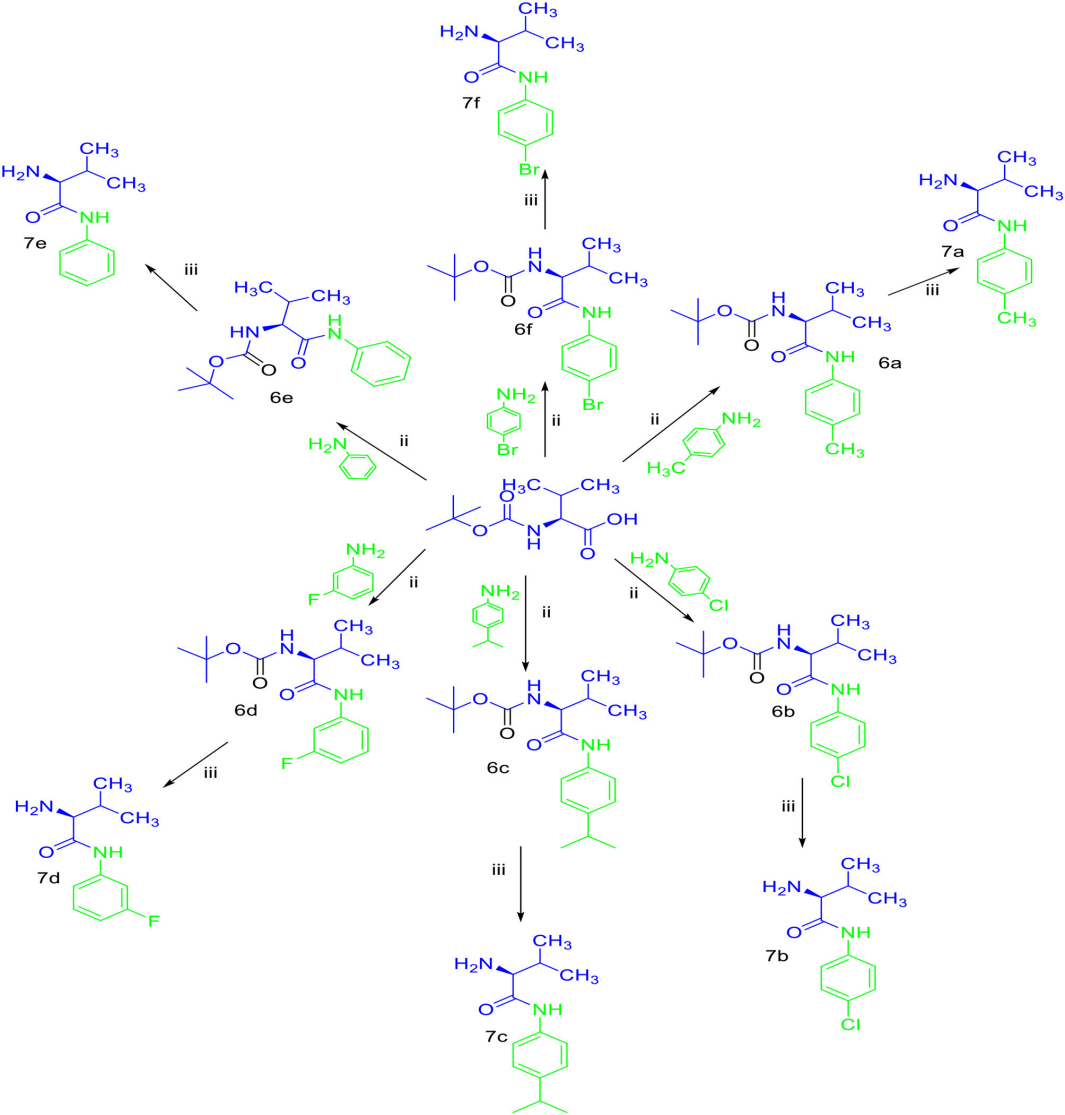
Synthesis of carboxamide derivatives (7a-f).

**Scheme 3:**
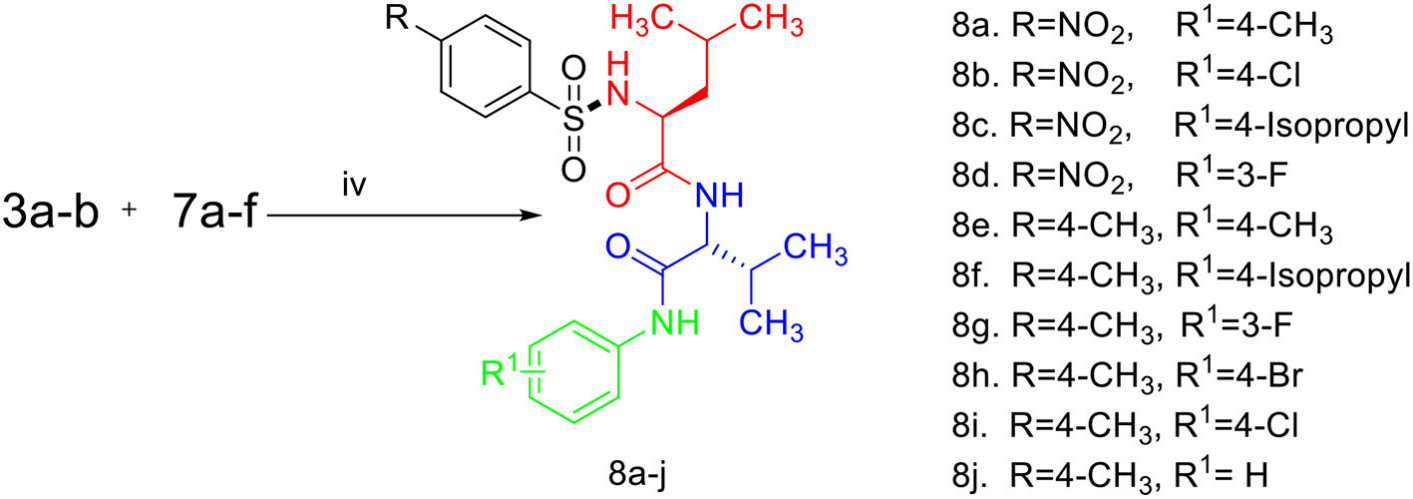
Synthesis of compounds (8a-j). (i) Na_2_CO_3_, H_2_O, HCl, −5–0°C, r.t, 4 h. (ii) EDC.HCl, HOBt, TEA, DCM, r.t, 19–24 h. (iii) TFA/DCM (1:1%). (iv) EDC.HCI, HOBt, TEA, r.t, 19–24 h.

Sulfonamides and peptides are two important pharmacophores as found in the literature and as such are sorted after functionalities in the drive to combat drug resistance by organisms. The reported compounds showed good binding interaction in the active site of the target proteins as shown in Figures 1, 2. To synthesize the compounds (**8a-j**) we adopted the use of classical peptide coupling reagent, 1-hydroxybenzotriazole (HOBt) and 1-ethyl-3-(3′-dimethylaminopropyl) carbodiimide hydrochloride (EDC.HCl) in the amidation of compounds (**7a-e**) with substituted benzenesulfonamides derived from L-leucine. Also, the activation of the carboxylic acid group of leucine was enhanced with HOBt as the EDC.HCI alone could not activate the carboxylic acid functionality. The use of HOBt and EDC.HCl was also recommended to reduce the risk of racemization. In our work, we synthesized and characterized molecules containing sulfonamide, carboxamide, and Dipeptides moieties. The reaction of substituted benzenesulfonyl chloride (**1a-b**) with L-leucine afforded substituted benzenesulphonamoyl alkanamides (**3a-b**) in Scheme 1. The reaction of commercially available Boc-protected valine with substituted amines using EDC.HCl, HOBt, and triethylamine (TEA) in DCM afforded the carbamate derivatives of valine (**6a-e**) in Scheme 2. Compounds (**7a-e**) were synthesized through the reaction of compound (**6a-e**) with DCM/ TFA (1:1%) for 1 h, respectively. The amidation of compound (**3a-b**) with the TFA salt of unprotected amides (**7a-e**) using peptide coupling reagents EDC.HCl, HOBt, TEA, to afford the desired products (**8a-j**) in Scheme 3. In the Infrared (IR) spectrum of **8a**, bands between ~3,312 and ~3,194 cm^−1^ are for N-H while ~1,642 and ~1,640 cm^−1^ are for the two carbonyls of amide, respectively. In the ^1^H-Nuclear magnetic resonance (NMR) spectrum of **8a**, the methylene group of leucine displayed a multiplet at δ1.30–1.41 and –methane group of leucine and valine showed multiplet at δ3.95–4.04 due to the interactions with the near amide group protons. The typical NH resonance of the sulfonamide part of the dipeptide conjugates was detected at the δ8.43 ppm region as a doublet peak. The other two NH resonances of the diamide were observed at δ9.91 ppm and 8.12 ppm as singlet and doublet, respectively. The aromatic protons were observed at δ8.33, 8.03 pp, and 7.44 ppm, 7.09 ppm region as doublet and doublet peaks, respectively, for the eight aromatic protons. In the ^13^C-Nuclear magnetic resonance (NMR) spectrum, two peaks at 169.85 and 171.21 ppm for the carbonyl carbons of the amide groups, eight peaks ranging from 119.62 to 149.73 ppm for aromatic carbons, and 10 peaks ranging from 18.69 to 58.78 ppm for aliphatic carbons confirmed the formation of **8a**, which was also supported by its high-resolution mass spectrometer (HRMS) peak spectrum with a peak at *m/z* 505.2125 for [M+H]^+^. All other compounds were in agreement with their structures.

### Physicochemical Properties Results

The pharmacokinetic properties were evaluated in the form of hydrogen bond donor ≤5, hydrogen bond acceptor ≤10, molecular weight value of ≤500, and partition coefficient (Log P) value ≤5. The results (Table 1) showed that the compounds would not pose oral bioavailability, transport, and permeability problems.

### Molecular Docking Results

**Figure 3:**
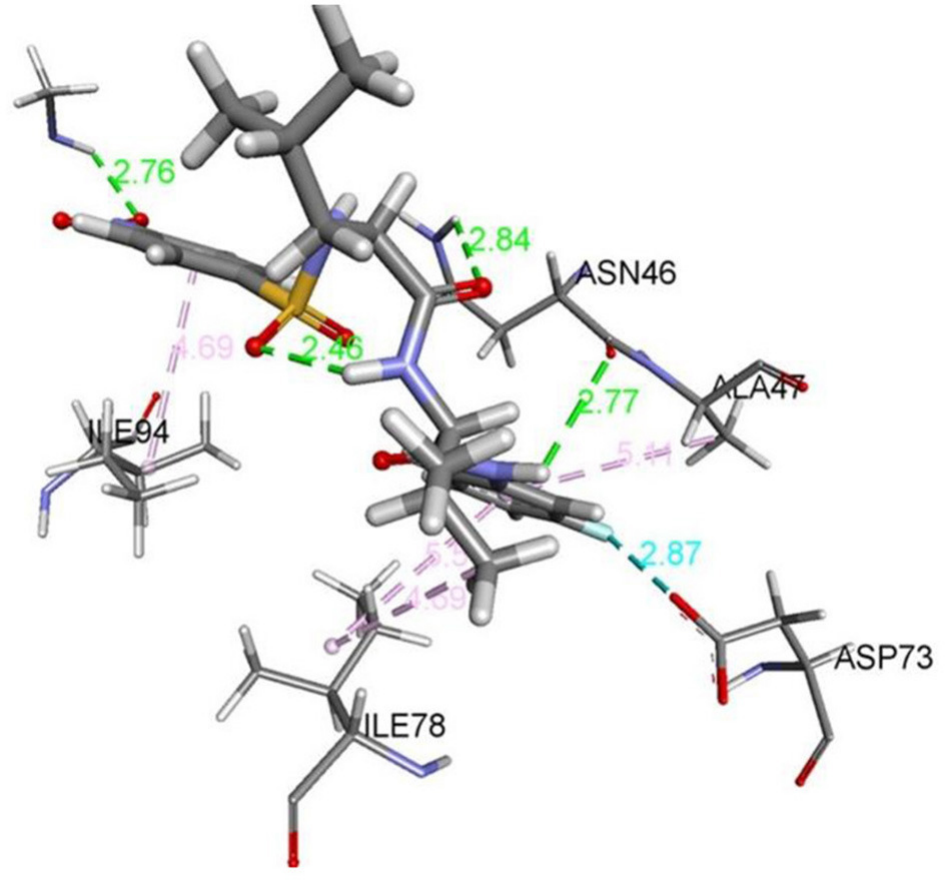
The stereo view of compound 8d in the binding cavity of 5MMN.

**Figure 4:**
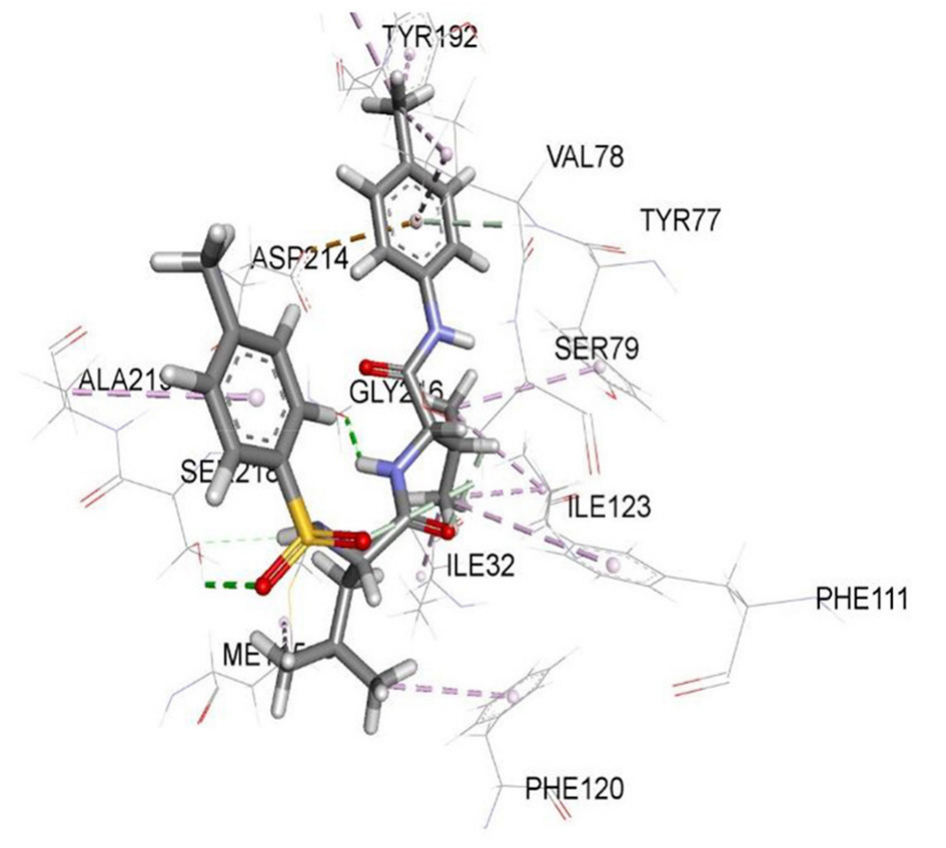
The stereo view of compound 8e in the binding cavity of 1SME.

The data in Table 2 reveals the binding energy of the synthesized dipeptides docked into the binding sites of the 5MMN and 1SME. The binding affinities of the leu-val dipeptides with the receptors were significant when compared to the ciprofloxacin and Chloroquine, respectively. Compounds **8d** and **8e** exhibited similar *in silico* antibacterial (−7.10 kcal/mol) and *in silico* antimalarial activity (−8.71 kcal/mol) as the standard (ciprofloxacin) (−5.38 kcal/mol) and Chloroquine (−6.11 kcal/mol), respectively. We moved extra to gain perception into the character of the binding interactions between the compounds and the receptors. Figures 3, 4 shows the stereo view of compounds **8d** and **8e** in the binding cavity of 5MMN and 1SME, while Figures 1, 2 illustrate how the atoms of compounds **8d** and **8e** interacted with the amino acid residues of 5MMN and 1SME, respectively. The binding interaction of **8d** with 5MMN in the active site is shown in Figures 1, 3. The O-atom of **8d** interacts with HD22 ASN 46 at an intermolecular distance of 2.84 Å. Another O-atoms interacted with H of NME 99 (2.76 Å), HA of ASN 46 at 2.70 Å, and with HH31 of NME 99 at an intermolecular distance of 2.84 Å. There is hydrogen bond interaction with O-atom of **8d** at 2.46 Å, while another H-atom interacted with O of ASN 46 (2.77 Å). There was an F-atom of compound **8d** interaction with OD2 of ASP 73 (2.87 Å).

**Table 2:** Binding free energy, ΔG (kcal/mol).

		**Antibacterial**	**Antimalarial**
**S/N**	**Compound code**	**5MMN: ΔG (kcal/mol) Scoring function: *London dG***	**1SME: ΔG (kcal/mol) Scoring function: *London dG***
1	8a	−6.57	−7.73
2	8b	−6.45	−7.69
3	8c	−6.71	−7.80
4	8d	−7.10	−7.01
5	8e	−6.05	−8.71
6	8f	−5.74	−7.75
7	8g	−6.31	−7.44
8	8h	−6.04	−7.45
9	8i	−6.70	−8.27
10	8j	−6.66	−7.06
11	Standard drugs	−5.38	−6.11

There were five hydrogen bonds formed in this interaction. In Figure 2, different atoms of compound **8e** interacted with the ISME receptor. O-atom of compound **8e** interacted with HG of SER 218, GLY 216, HB2 of SER 79, and HB3 of SER 79 at an intermolecular distance of 2.79, 2.46, 3.07, and 2.55 Å, respectively, through hydrogen bond interaction. Also, compound **8e** through pi donor hydrogen bond interaction had contact with H VAL 78 at a distance of 3.21 Å. Other hydrophobic interactions with **8d**, **8e**, and conserved amino acid residues were also shown in Figures 1, 2, respectively.

Table 3 further validated the docking protocols and provided insights on the nature of the binding interactions of both the native ligands and the hit molecules with the amino acid residues in the binding sites of the receptors. Here the major amino acid residues involved in the interactions were considered. In 1SME receptor, both the native ligand (pepstatin) and compound 8e interacted with almost the same amino acide residues in its binding site. For example, pepstatin and 8e interacted with GLY 216 through H-bonding at distances of 3.75 and 2.46 Å, respectively. This further shows that the compound 8e occupied the same binding site as the native ligand, thereby validating the docking. We also noted that 8e was closer to interact with GLY 216 than the native ligand. This probably could have resulted in the higher binding affinity of 8e. This trend was also observed with most of the amino acid residues interacting with the molecules.

**Table 3:** Comparison of binding interactions of the native ligands and hit molecule (8e) to the binding sites of 1SME and 5MMN.

	**Amino acid residue**	**Type of interaction**	**Distance of interaction (Å)**
1SME-native ligand (Pepstatin)	TYR 192 GLY 216 PHE 111 SER 218 ILE 290 VAL 78	H-Bonding H-Bonding Pi-alkyl H-Bonding Pi-alkyl H-Bonding	5.89 3.75 6.24 3.66 4.74 3.74
1SME-8e	GLY 216 TYR 192 TYR 192 PHE 111 PHE 120 ASP 214	H-Bonding Pi-alkyl Pi-alkyl Pi-alkyl Pi-alkyl Pi-anion	2.46 4.99 5.03 5.47 5.37 7.39
5MMN-native ligand: 1-ethyl-3-[8-methyl-5-(2-methyl-pyridin-4-yl)-isoquinolin-3-yl]-urea	THR 165 ASP 73 ASP 73 GLY 77 ILE 94 ILE 78 ILE 78 PRO 79	H-bonding H-bonding H-bonding Amide-pi stacked Pi-alkyl Pi-alkyl Pi-alkyl Pi-alkyl	4.80 3.92 4.52 5.83 5.38 4.86 4.53 5.09
8d-5MMN	ILE 94 ILE 78 ILE 78 ASN 46 ASN 46 ALA 47 ASP 73	Pi-alkyl Pi-alkyl Pi-alkyl H-bonding H-bonding Pi-alkyl Fluorine	4.58 5.37 4.50 3.27 4.53 4.97 4.63

### *In vitro* Antimicrobial Activities

Table 4 comprises of minimum inhibitory concentration (MIC) of the synthesized compounds. The *in vitro* antibacterial properties of the compounds and Ciprofloxacin were evaluated against (*staphylococcus aureus* and *Bacillus subtilis*) and (*Salmonella typhi* and *Escherichia coli*) by the agar dilution method as Gram-positive and Gram-negative organisms, respectively. The *in vitro* antifungal properties of leu-val dipeptides synthesized were deduced by the agar dilution method against two fungal strains (*Candida albicans* and *Aspergillus niger*) and fluconazole as reference drug. The figures in Table 4 revealed that compound **8a** with MIC value of **1.2** **×** **10**^**−3**^ M displayed comparable anti-bacterial activity against *S. aureus* to standard (9.1 × 10^−4^ M). Compounds **8a, 8b, 8g**, and **8j** with MIC values ranging from (5.7 × 10^−4^M−8.4 × 10^−4^ M) have the same or comparable activity with the standard drug (9.1 × 10^−4^ M) against *B. subtilis*. All the compounds showed inhibition against these two Gram-positive organisms except compounds **8f** and **8h**, which are resistant to *S. aureus*. For the Gram-negative bacteria, it was revealed compounds **8b** with MIC value of 9.5 × 10^−4^ M showed activity against *E. coli*. All other compounds inhibits the growth of *E. coli* though less than the standard.

**Table 4:** Minimum inhibitory concentration (MIC) in molar concentration.

**Compound number**	***S. aureus***	***B. subtilis***	***E.coli***	***Sal. typhi***	***C. albicans***	***Asp. niger***
8a	1.2 × 10^−3^	6.0 × 10^−4^	1.4 × 10^−3^	1.2 × 10^−3^	1.8 × 10^−3^	1.6 × 10^−3^
8b	1.1 × 10^−3^	5.7 × 10^−4^	9.5 × 10^−4^	1.1 × 10^−3^	1.7 × 10^−3^	1.3 × 10^−3^
8c	1.7 × 10^−3^	1.3 × 10^−3^	1.7 × 10^−3^	1.9 × 10^−3^	1.3 × 10^−3^	1.7 × 10^−3^
8d	1.4 × 10^−3^	9.8 × 10^−4^	1.6 × 10^−3^	1.2 × 10^−3^	1.6 × 10^−3^	1.6 × 10^−3^
8e	1.5 × 10^−3^	1.3 × 10^−3^	2.1 × 10^−3^	1.9 × 10^−3^	+	+
8f	+	1.6 × 10^−3^	2.0 × 10^−3^	+	+	+
8g	2.1 × 10^−3^	8.4 × 10^−4^	1.5 × 10^−3^	1.9 × 10^−3^	2.1 × 10^−3^	+
8h	+	1.3 × 10^−3^	1.8 × 10^−3^	+	1.3 × 10^−3^	1.8 × 10^−3^
8i	1.6 × 10^−3^	1.2 × 10^−3^	1.6 × 10^−3^	2.0 × 10^−3^	1.6 × 10^−3^	1.6 × 10^−3^
8j	1.7 × 10^−3^	6.5 × 10^−4^	2.0 × 10^−3^	1.7 × 10^−3^	+	+
Cipro.	9.1 × 10^−4^	9.1 × 10^−4^	9.1 × 10^−4^	9.1 × 10^−4^	+	+
Fluco.	+	+	+	+	9.8 × 10^−4^	9.8 × 10^−4^

The data in Table 4 revealed that the synthesized compounds showed good activity against *S. typhi* though less than the standard except for compounds **8f** and **8h** that are resistant to *S. typhi*. Further evaluation of the data revealed that compounds **8a** and **8b** are more potent as antibacterial agents when compared with other synthesized leu-val dipeptides derivatives. More so, it was revealed that the synthesized compounds showed better activity against *C. albicans* and *A. niger* though less active when compared with fluconazole except for compounds **8e, 8f**, and **8j** that do not have any inhibition against *C. albicans* and compounds **8e**, **8f**, **8g**, and **8j** that are resistant against *A. niger*.

### *In vivo* Antimalarial

The leu-val dipeptides synthesized were evaluated for *in vivo* antimalarial activity against *P*. *berghei* NK (65 Strain) poison mice. The animal ethics committee, Veterinary Medicine Department, University of Nigeria, Nsukka gave permission and approval for the use of animals in this experiment (PG/PhD/16/80697). The percentage inhibition of parasite was calculated from the equation [(A-B)/A] × 100 (Ugwuja et al., 2019; Ezugwu et al., 2020); where A = parasitemia of the untreated group and B = parasitemia of the tested group. In this study, the compounds with the percentage inhibition below 30 are inactive, 30–40 are partially active, and 40 and above are regarded to be active. From Table 5 it was revealed that compounds **8a**, **8b**, **8d**, **8e**, **8g**, **8h**, **8i**, and **8j** which have 43.30–61.90% inhibition were active in comparable with the standard drug (with 67% inhibition). The analysis evaluation of the compounds synthesized revealed that compound **8j** (61.90%) was the most potent antimalarial when compared with others, looking at the structure-activity relationship on the 4-nitrophenylsulfonamide hybrids (**8a**-**d**). The effect of 4-methyl, 4-chloro, 4-Isopropyl, and 3-fluoro substituent on the *N-*phenylacetamide was studied, and it was revealed that 4-methyl-*N*-phenylacetamide derivative (**8a**, 60.2%) was the most potent inhibitor, for *P. berghei* followed by **8b** (57.8%) and **8d** (43.4%), and **8c** were considered moderately active. The effects of 4-methyl, 4-bromo, 4-chloro, 4-Isopropyl, and 3-fluoro substituent on the *N*-phenylacetamide among *p*-methylbenzenesulfonamide hybrids (**8d**-**j**) revealed that compounds **8j** and **8h** with 61.90 and 49.80% inhibition, respectively, were more active.

**Table 5:** Percentage inhibition of parasite in mice.

**Compounds no**.	**% parasitaemia before treatment**	**% Parasitaemia after treatment**	**% inhibition**
8a	67.0 ± 1.52753	33.0 ± 10.01665	60.2 ± 12.07159
8b	59.7 ± 3.38296	23.0 ± 2.08167	57.8 ± 15.26055
8c	58.3 ± 2.33333	51.7 ± 12.83658	37.8 ± 15.47055
8d	68.7 ± 3.92994	47.0 ± 3.21455	43.4 ± 3.88344
8e	69.3 ± 1.45297	38.0 ± 4.00000	43.0 ± 14.27737
8f	63.0 ± 5.33333	74.3 ± 4.40959	10.4 ± 5.32301
8g	65.0 ± 1.52753	45.0 ± 11.93035	45.8 ± 14.37370
8h	65.7 ± 4.97773	42.3 ± 5.48736	49.0 ± 6.61421
8i	63.3 ± 5.20683	48.3 ± 7.96520	41.8 ± 9.59103
8j	55.3 ± 2.96273	31.7 ± 5.78312	61.9 ± 6.96799
Arte.	62.0 ± 0.57735	27.0 ± 1.76383	67.1 ± 2.11660
NTC	68.0 ± 1.73205	83.0 ± 1.73205	0.0 ± 0.0000

*Arte, Artemisinin; NTC, Non Treated Control*.

*Values are means of three determinations ± SEM*.

## Conclusion

In this paper, we have described an approach to obtain leu-val based dipeptide derivatives that were tested for their antimalarial and antimicrobial properties. The results obtained showed that among the leu-val dipeptides synthesized, compound **8j** was more active against *P. berghei*. Compound **8b** was the most to inhibit growth of *E. coli*, compound **8a** and **8b** were most active against *S. aureus*, compounds **8a, 8b**, and **8d** were most active against *S. typhi, B. subtilis* is inhibited most with compounds **8a, 8b**, and **8j**. Compounds **8c, 8h**, and **8b** were the most active synthesized compounds against *C. albicans* and *A. niger*, respectively.

## Data Availability Statement

The datasets presented in this study can be found in online repositories. The names of the repository/repositories and accession number(s) can be found in the article/Supplementary Material.

## Ethics Statement

The animal study was reviewed and approved by the Animal Ethics Committee, Veterinary Medicine Department, University of Nigeria, Nsukka.

## Author Contributions

All authors listed have made a substantial, direct and intellectual contribution to the work, and approved it for publication.

## Conflict of Interest

The authors declare that the research was conducted in the absence of any commercial or financial relationships that could be construed as a potential conflict of interest.

## References

[B1] Abdelaziz A. M. Yu M. Li P. Zhong L. Singab A. N. B. Hanna A. G. (2015). Synthesis and evaluation of 5-chloro-2-methoxy-*N*-(4-sulphamoylphenyl) benzamide derivatives as anti-cancer agents. Med. Chem. 5, 253–260. 10.4172/2161-0444.1000272

[B2] Amit M. Rahul P. G. Sunil B. Nathan E. G. Ben M. D. Abhay T. S. (2015). Antiplasmodial activity of short peptide-based compounds. RSC Adv. 5:22674 10.1039/C5RA00779H

[B3] Day T. Greenfield S. A. (2004). Bioactivity of a peptide derived from acetylcholinesterase in hippocampal organotypic cultures. Exp. Brain Res. 155:500. 10.1007/s00221-003-1757-1 14685807

[B4] Deniz Ü. P. Zehra Küçükbay F. Küçükbay H. Angeli A. Supuran C. T. (2019). Synthesis carbonic anhydrase enzyme inhibition and antioxidant activity of novel benzothiazole derivatives incorporating glycine, methionine, alanine, and phenylalanine moieties. J. Enzyme Inhib. Med. Chem. 34:343349 10.1080/14756366.2018.1553040 PMC6327993 30734592

[B5] Eze F. U. Okoro U. C. Ugwu D. I. Okafor S. N. (2019). Biological activity evaluation of some new benzenesulphonamide derivatives. Front. Chem. 7:634 10.3389/fchem.2019.00634 31620427 PMC6759663

[B6] Ezugwu J. A. Okoro U. C Ezeokonkwo M. A Bhimapaka C. Okafor S. N. Ugwu D. I.. (2020). Synthesis and biological evaluation of Val–Val dipeptide–sulfonamide conjugates. Arch. Pharm. 353:e2000074. 10.1002/ardp.202000074 32390214

[B7] Jatinder P. K. G. Singh S. Sethi N. (2015). Synthesis, characterization and antimicrobial activity ofprotected dipeptides and their deprotected analogs. Orient. J. Chem. 31, 417–421. 10.13005/ojc/310149

[B8] Jiao Z. G. He H. Q. Zeng C. C. Tan J. J. Hu L. M. Wang C. X. (2010). Design, synthesis and anti-HIV integrase evaluation of *N*-(5-chloro-8-hydroxy-2Styrylquinolin-7-yl) benzenesulfonamide derivatives. Molecules 15, 1903–1917. 10.3390/molecules15031903 20336021 PMC6257356

[B9] Jyothi B. Madhavi N. (2018). Synthesis and biological screening of pyrimidine linked benzene sulfonamide derivatives. Int. J. Pharm. Sci. Res. 9, 5534–5543. 10.13040/IJPSR.0975-8232

[B10] Kayser H. Meisel H. (1996). Stimulation of human peripheral blood lymphocytes by bioactive peptides derived from bovine milk proteins. FEBS Lett. 383, 18–20. 10.1016/0014-5793(96)00207-4 8612782

[B11] Khavinson V. K. Anisimov V. N. (2000). Synthetic dipeptide vilon (LLys-L-Glu) increases life span and inhibits a development of spontaneous tumors in mice. Doklady Akad. Nauk. 372, 421–423. 10944717

[B12] Kilicaslan S. Arslan M. Ruya Z. Bilen C. Adem E. Nahit G.. (2016). Synthesis and evaluation of sulfonamide-bearing thiazole as carbonic anhydrase isoforms hCA I and hCA II. J. Enzyme Inhib. Med. Chem. 31, 1300–1305. 10.3109/14756366.2015.1128426 26744900

[B13] Kitts D. D. Weiler K. (2003). Bioactive proteins and peptides from food sources. Applications of bioprocesses used in isolation and recovery. Curr. Pharm. Design 9, 1309–1323. 10.2174/1381612033454883 12769739

[B14] Küçükbay H. Bugday N. Küçükbay F. Z. Berrino E. Bartolucci G. Del Prete S.. (2019). Synthesis and carbonic anhydrase inhibitory properties of novel 4*-*(2-aminoethyl)benzenesulphonamide-dipeptide conjugates. Bioorg. Chem. 83, 414–423. 10.1016/j.bioorg.2018.11.003 30419497

[B15] Kumar V. Mudgal M. M. Rani N. Jha A. Jaggi M. Singh A. T.. (2009). Synthesis of functionalized amino acid derivatives as new pharmacophores for designing anticancer agents. J. Enzyme Inhib. Med. Chem. 24, 763–770. 10.1080/14756360802362975 18720190

[B16] Maloy Kumar P. Panda G. Srivastava K. Puri S. K. (2018). Design, synthesis and antimalarial activity of benzene and isoquinoline sulfonamide derivatives. Bioorg. Med. Chem. Lett. 18, 776–781. 10.1016/j.bmcl.2007.11.038 18039570

[B17] Montalbetti C. A. G. N. Falque V. (2005). Amide bond formation and peptide coupling. Tetrahedron 61:10852 10.1016/j.tet.2005.08.031

[B18] Nitta A. Nishioka H. Fukumitsu H. Furukawa Y. Sugiura H. Shen L.. (2004). Hydrophobic dipeptide Leu-Ile protects against neuronal death by inducing brainderived neurotrophic factor and glial cell line-derived neurotrophic factor synthesis. J. Neurosci. Res. 78, 250–258. 10.1002/jnr.20258 15378610

[B19] Okokon J. E. Nwafor P. A. (2009). J Ethnopharmacol. 121:74. 10.1016/j.jep.2008.09.034 18996464

[B20] Panchaud P. Bruyère T. Blumstein A. C. Bur D. Chambovey A. Ertel E. A.. (2017). Discovery and optimization of isoquinoline ethyl ureas as antibacterial agents. J. Med. Chem. 60, 3755–3775. 10.1021/acs.jmedchem.6b01834 28406299

[B21] Panda S. S. Ibrahim M. A. Küçükbay H. Meyers M. J. Sverdrup F. M. El-Feky S. A.. (2013). Synthesis and antimalarial bioassay of quinine – peptide conjugates. Chem. Biol. Drug Des. 82:361. 10.1111/cbdd.12134 23497252

[B22] Qadir M. A. Ahmed M. Aslam H. Waseem S. Shafiq M. I. (2015). Amidine sulfonamides and benzene sulfonamides: synthesis and their biological evaluation. J. Chem. 15:8 10.1155/2015/524056

[B23] Qi Z. Verma R. Gehring C. Yamaguchi Y. Zhao Y. Ryan C. A.. (2010). Ca2þ signaling by plant Arabidopsis thaliana pep peptides depends on AtPepR1, a receptor with guanylyl cyclase activity, and cGMPactivated Ca2þ channels. Proc. Natl. Acad. Sci. U.S.A. 107:21193 10.1073/pnas.1000191107 21088220 PMC3000296

[B24] Silva A. M. Lee A. Y. Gulnik S. V. Maier P. Collins J. Bhat T. N.. (1996). Structure and inhibition of plasmepsin II, a hemoglobin-degrading enzyme from *Plasmodium falciparum*. Proc. Natl. Acad. Sci. U.S.A. 93:10034. 10.1073/pnas.93.19.10034 8816746 PMC38331

[B25] Thompson A. A. Liu W. Chun E. Katritch V. Wu H. Vardy E.. (2012). Structure of the nociceptin/ orphanin FQ receptor in complex with a peptide mimetic. Nature 485:495. 10.1038/nature11085 22596163 PMC3356928

[B26] Ugwu D. I. Ezema B. E. Eze F. U. Ugwuja D. I. (2014). Synthesis and structural activity relationship study of antitubercularcarboxamides. Int. J. Med. Chem. 2014, 1–18. 10.1155/2014/614808 25610646 PMC4295614

[B27] Ugwu D. I. Okoro U. C. Mishra N. K. (2018a). Synthesis, characterization and anthelmintic activity evaluation of pyrimidine derivatives bearing carboxamide and sulphonamide moieties. J. Serbian Chem. Soc. 83, 401–409. 10.2298/JSC170127109U

[B28] Ugwu D. I. Okoro U. C. Mishra N. K. (2018b). Synthesis, characterization and in vivo antitrypanosomal activities of new carboxamides bearing quinoline moiety. PLoS ONE 13:e0191234 10.1371/journal.pone.0191234 29324817 PMC5764481

[B29] Ugwu D. I. Okoro U. C. Ukoha P. O. Gupta A. Okafor S. N. (2018c). Novel anti-inflammatory and analgesic agents: synthesis, molecular docking and *in vivo* studies. J. Enzyme Inhib. Med. Chem. 33, 405–415. 10.1080/14756366.2018.1426573 29372659 PMC7011796

[B30] Ugwuja D. I. Okoro U. C. Soman S. S. Soni R. Okafor S. N. Ugw D. I.. (2019). New peptide derived antimalaria and antimicrobial agents bearing sulphonamide moiety. J. Enzyme Inhib. Med. Chem. 34, 1388–1399. 10.1080/14756366.2019.1651313 31392901 PMC6713104

[B31] Zehra Küçükbay F. Küçükbay H. Tanc M. Supuran C. T. (2016). Synthesis and carbonic anhydrase I, II, IV and XII inhibitory properties of N-protected amino acid– sulfonamide conjugates. J. Enzyme Inhib. Med. Chem. 31, 1476–83. 10.3109/14756366.2016.1147438 26899532

