# Supplementary material for: Novel Leu-Val Based Dipeptide as Antimicrobial and Antimalarial Agents: Synthesis and Molecular Docking
Source: Front Chem. 2020 Nov 24;8:583926. doi: 10.3389/fchem.2020.583926 (PMC7732421; doi:10.3389/fchem.2020.583926)
Supplement: Supplementary file 1 [file Data_Sheet_1.docx]

**SUPPORTING DOCUMENT**

**Antimicrobial Evaluation**

The microorganisms below were isolated clinically from University of Nigeria, Nsukka at Department of Pharmaceutical Microbiology and Biotechnology laboratory**.** S*taphylococcus aureus, Escherichia coli, Bacillus subtilis, Salmonella typhi, Candida albicans,* and *Aspergillus niger*.

**Standardization of the test organism suspension**: The standardization of the microorganisms were done using 0.5 MacFaland turbid equivalent.

**Control test (standard)**: The standard antibiotics used were ciprofloxacin and fluconazole.

**Experimental**:

4.0ml of suspension of stock concentration 50mg/ml was transferred to the sterile Petri dish, a 16.0ml volume of double strength sterile molten agar was transferred to the same plate to mix uniformly thus, 1mg/ml concentration was obtained. The other concentrations 0.9mg/ml, 0.8mg/ml, 0.7mg/ml, 0.6mg/ml, 0.5mg/ml, 0.4mg/ml, 0.3mg/ml, 0.2mg/ml, 0.1mg/ml, were obtained using the same C_1_V_1_=C_2_V_2_ formula. The molten agar plates with different concentrations of the compounds were allowed to gel. The plates were divided into seven equal parts with permanent marker. The test microbes were patterned on the plates, labeled and kept in an incubator at 37 ^O^C for 24 h and 35 ^O^C for 48 h respectively for antibacterial and antifungal activities. Further incubation of the plates for 24 h at 37 ^O^C and 48 h at 25 ^O^C respectively used to test for bactericidal and fungicidal activities.

***In vivo* anti-malaria test**

**Experimental Design and Treatment of Mice**

Methods reported by (Okokon, J.E, and Nwafor, P.A, 2009 and Ezugwu *et al*., 2020) for antiplasmodial assay against Plasmodium berghei infection in mice were adopted. About forty eight infected mice were randomly divided into twelve groups, each having four mice. A stock of parasitized erythrocytes was obtained from infected mice, with a minimum peripheral parasitemia of 20 % by cardiac puncture in EDTA-coated tube. The percentage parasitaemia was obtained by counting the number of parasitized red blood cells against the total number of red blood cells. The cell concentration made from the stock was set and diluted with physiological saline such that 0.2 mL of the final inoculum contained 1 x10^7^ parasitized red blood cells which are the standard inoculums for the infection of a single mouse. After 7 days of infection, animals begin to receive treatment (50 mg/kg) of the synthesized compounds (8a–8j) for 7 days with constant check of the percentage of parasitemia after a 4-day interval. Artemisinin (50 mg/kg body weight.) was given to the other mice in group twelve as positive control, group thirteen was not treated. All the compounds and the drugs were given orally by using a standard intragastric tube.

Spectra of compounds 8a-j

**^1^H NMR spectra of (*S*)-*N*-({(*S*)-1-[(4-Methylphenyl)amino]-3-methyl-1-oxobutan-2-yl})-4-methyl-2-(4-nitrophenylsulfonamido) pentanamide (8a)**

**^13^C NMR spectra of (*S*)-*N*-({(*S*)-1-[(4-Methylphenyl)amino]-3-methyl-1-oxobutan-2-yl})-4-methyl-2-(4-nitrophenylsulfonamido) pentanamide (8a)**

**^1^H NMR spectra of (*S*)-*N*-({(*S*)-1-[(4-Chlorophenyl)amino]-3-methyl-1-oxobutan-2-yl})-4-methyl-2-(4-nitrophenylsulfonamido) pentanamide (8b)**

**^13^C NMR spectra of (*S*)-*N*-({(*S*)-1-[(4-Chlorophenyl)amino]-3-methyl-1-oxobutan-2-yl})-4-methyl-2-(4-nitrophenylsulfonamido) pentanamide (8b)**

**^1^H NMR spectra of (*S*)-*N*-({(*S*)-1-[(4-Isopropylphenyl)amino]-3-methyl-1-oxobutan-2-yl})-4-methyl-2-(4-nitrophenyl sulfonamido) pentanamide (8c)**

**^13^C NMR spectra of (*S*)-*N*-({(*S*)-1-[(4-Isopropylphenyl)amino]-3-methyl-1-oxobutan-2-yl})-4-methyl-2-(4-nitrophenyl sulfonamido) pentanamide (8c)**

**^1^H NMR spectra of (*S*)-*N*-({(*S*)-1-[(3-Fluorophenyl)amino]-3-methyl-1-oxobutan-2-yl})-4-methyl-2-(4-nitrophenylsulfonamido) pentanamide(8d)**

**^13^C NMR spectra of (*S*)-*N*-({(*S*)-1-[(3-Fluorophenyl)amino]-3-methyl-1-oxobutan-2-yl})-4-methyl-2-(4-nitrophenylsulfonamido) pentanamide(8d)**

**^1^H NMR spectra of (*S*)-*N*-({(*S*)-1-[(4-Methylphenyl)amino]-3-methyl-1-oxobutan-2-yl})-4-methyl-2-(4-methylphenylsulfonamido) pentanamide (8e)**

**^13^C NMR spectra of (*S*)-*N*-({(*S*)-1-[(4-Methylphenyl)amino]-3-methyl-1-oxobutan-2-yl})-4-methyl-2-(4-methylphenylsulfonamido) pentanamide (8e)**

**^1^H NMR spectra of (*S*)-*N*-({(*S*)-1-[(4-Isopropylphenyl)amino]-3-methyl-1-oxobutan-2-yl})-4-methyl-2-(4-methylphenyl sulfonamido) pentanamide (8f)**

**^13^C NMR spectra of (*S*)-*N*-({(*S*)-1-[(4-Isopropylphenyl)amino]-3-methyl-1-oxobutan-2-yl})-4-methyl-2-(4-methylphenyl sulfonamido) pentanamide (8f)**

**^1^H NMR spectra of (*S*)-*N*-({(*S*)-1-[(3-Fluorophenyl)amino]-3-methyl-1-oxobutan-2-yl})-4-methyl-2-(4-methyl-phenyl sulfonamido) pentanamide (8g)**

**^13^C NMR spectra of (*S*)-*N*-({(*S*)-1-[(3-Fluorophenyl)amino]-3-methyl-1-oxobutan-2-yl})-4-methyl-2-(4-methyl-phenyl sulfonamido) pentanamide (8g)**

**^1^H NMR spectra of (*S*)-*N*-({(*S*)-1-[(4-Bromophenyl)amino]-3-methyl-1-oxobutan-2-yl})-4-methyl-2-((4-methyl-phenyl)sulfonamido)pentanamide(8h)**

**^13^C NMR spectra of (*S*)-*N*-({(*S*)-1-[(4-Bromophenyl)amino]-3-methyl-1-oxobutan-2-yl})-4-methyl-2-((4-methyl-phenyl)sulfonamido)pentanamide(8h)**

**^1^H NMR spectra of (*S*)-*N*-({(*S*)-1-[(4-Chlorophenyl)amino]-3-methyl-1-oxobutan-2-yl})-4-methyl-2-((4-methyl-phenyl)sulfonamido)pentanamide (8i)**

**^13^C NMR spectra of (*S*)-*N*-({(*S*)-1-[(4-Chlorophenyl)amino]-3-methyl-1-oxobutan-2-yl})-4-methyl-2-((4-methyl-phenyl)sulfonamido)pentanamide (8i)**

**^1^H NMR spectra of (*S*)-*N*-({(*S*)-1-[(Phenyl)amino]-3-methyl-1-oxobutan-2-yl})-4-methyl-2-**

**(4-methylphenylsulfonamido) pentanamide (8j)**

**^13^C NMR spectra of (*S*)-*N*-({(*S*)-1-[(Phenyl)amino]-3-methyl-1-oxobutan-2-yl})-4-methyl-2-**

**(4-methylphenylsulfonamido) pentanamide (8j)**
